# Developing a Simple and Feasible Process for the Crude Extraction of Livetins and Phosvitin from Egg Yolk

**DOI:** 10.3390/foods13243990

**Published:** 2024-12-10

**Authors:** Fan Zhang, Yongmei Ding, Zipei Zhang, Hangxin Zhu, Han Jiao, Shijian Dong, Junhua Li, Luping Gu, Cuihua Chang, Yanjun Yang, Yujie Su

**Affiliations:** 1 State Key Laboratory of Food Science and Resources, School of Food Science and Technology, Jiangnan University, Wuxi 214122, China; fanzhangouc@163.com (F.Z.); lijunhua@jiangnan.edu.cn (J.L.); guluping@jiangnan.edu.cn (L.G.); chang.cuihua@jiangnan.edu.cn (C.C.); yangyj@jiangnan.edu.cn (Y.Y.); 2 College of Science, Wuhan University of Science and Technology, Wuhan 430065, China; dingyongmei@wust.edu.cn; 3 Food Science Program, Division of Food, Nutrition & Exercise Sciences, University of Missouri, Columbia, MO 65211, USA; zz5hd@missouri.edu (Z.Z.); hzycc@missouri.edu (H.Z.); 4 Anhui Rongda Food Co., Ltd., Xuancheng 242200, China; qzjiaohan@163.com (H.J.); dsjwin@126.com (S.D.)

**Keywords:** egg yolk, livetins, phosvitin, low-density lipoprotein, high-density lipoprotein, separation

## Abstract

Due to imbalanced demand favoring egg whites, the egg industry faces a surplus of egg yolk, limiting overall growth. This study designed a feasible process for the crude extraction of livetins and phosvitin (PV) and revealed the related separation mechanisms. Our method utilized a 1:9 egg yolk dilution at pH 6.15–6.29, incubated at 4–7.5 °C, to reduce the dispersibility of lipoproteins in the water-soluble fraction (WSF). Adding 0.04–0.05% (*w*/*v*) sodium alginate to WSF at pH 5.40 effectively removed suspended low-density lipoprotein (LDL) through electrostatic complexation, increasing livetins electrophoretic bands from 51.90% to 91.04%. The dispersion of the high-density lipoprotein (HDL)-PV complex was jointly affected by NaCl and pH, with phosphocalcic bridges fully disrupted when NaCl concentration exceeded 7.5% (*w*/*v*). Na^+^ and Ca^2+^ were adsorbed onto the negatively charged protein surface at pH 5–8, inducing strong hydration repulsion, thereby resulting in the individual dispersion of HDL and PV. Based on the solubility difference in low ionic strength solutions at neutral pH, HDL could be effectively removed after dialysis, increasing PV electrophoretic bands from 8.45% to 61.50%. This simple and feasible separation process may provide a reliable foundation for further purification via membrane filtration and chromatography.

## 1. Introduction

Currently, there is a higher demand for egg whites due to their low-fat, high-protein composition, catering to health-conscious consumers and various food processing applications [1]. However, the imbalance in demand not only results in surplus egg yolk but also triggers a continuous compensatory increase in egg white prices, consequently stagnating the growth of the egg industry [2]. Developing new commodity products through the fractionation of proteins and their application in various food processing, as well as the development of pharmaceutical, nutraceutical, and cosmeceutical agents, have been reported as two key approaches to increase the value and utilization of egg yolk [3]. Egg yolk is mainly composed of 68% low-density lipoprotein (LDL), 16% high-density lipoprotein (HDL), 10% livetins, and 4% phosvitin (PV) on the basis of dry matter [4]. Livetins are water-soluble proteins consisting of α-livetin, β-livetin, and γ-livetin (IgY) in a ratio of 2:5:3 [5]. γ-Livetin is widely studied for its immune defense role and potential in passive immunotherapy and as an antibody alternative in research and therapy. PV is a natural phosphoprotein, in which 50–57% of the amino acids are serine, and 90% of them are phosphorylated [6]. As value-added proteins, livetins and PV have high potential in anti-inflammatory [7], antibacterial [8], antioxidant [9], and gut health improvement applications [10]. However, the presence of LDL and HDL in egg yolk, respectively, interfered with the separation of livetins and PV.

In general, egg yolk can be divided into “plasma and granules” through centrifugation or into “water-soluble fraction (WSF) and insoluble fraction (ISF)” by water dilution. However, regardless of the approach adopted, the following facts will be faced in further separation: (1) the suspension of amphiphilic LDL in plasma or WSF; (2) the presence of phosphocalcic bridges between PV and insoluble HDL in granules. These two facts make it difficult to separate hydrophilic livetins and PV from lipoproteins. High-purity livetins and PV can be prepared using membrane filtration [11] and chromatography [12], respectively. Nevertheless, both techniques have limitations in practical production. The high lipid content of LDL can easily cause membrane blockage during livetin separation, posing challenges for continuous production. Chromatography often struggles with lower processing capacity and higher costs. Therefore, obtaining crude extracts of livetins and PV through simple methods may provide a reliable foundation for further purification.

Universal methods for the crude separation of egg yolk proteins, such as salting-out [13], polymer precipitation [14], and selective denaturation [15], typically rely on disrupting the interactions between proteins and water. These nonspecific approaches often involve the use of substantial amounts of exogenous processing aids. As a result, separation efforts are generally confined to isolating a single component and are mostly limited to laboratory-scale applications [16]. Additionally, residues are often difficult to separate further or may become denatured during processing. Therefore, the differences in solubility, dispersion state, and density among egg yolk proteins under various conditions (e.g., temperature, pH, ionic strength, salt concentration, and their synergistic interactions) should be fully utilized [17]. In this study, a feasible process was designed to achieve the co-production of livetins and PV, and the key factors and underlying mechanisms affecting the crude separation were investigated. Our process only involved common unit operations such as stirring, refrigeration, centrifugation, and desalination. The processing aids were also readily available.

## 2. Materials and Methods

### 2.1. Materials and Chemicals

Fresh eggs were purchased from the local supermarket (Wuxi, China). Sodium alginate was obtained from Sinopharm Chemical Reagent Corp. Ltd. (Shanghai, China). Loading buffer (4×) was provided by Takara Biomedical Technology Co., Ltd. (Beijing, China). A premixed protein marker and calcium content assay kit (BC0725) were purchased from Solarbio Science and Technology Co., Ltd. (Beijing, China). All other chemicals used in this study were of analytical grade.

### 2.2. Isolation of Egg Yolk Components

**Figure 1:**
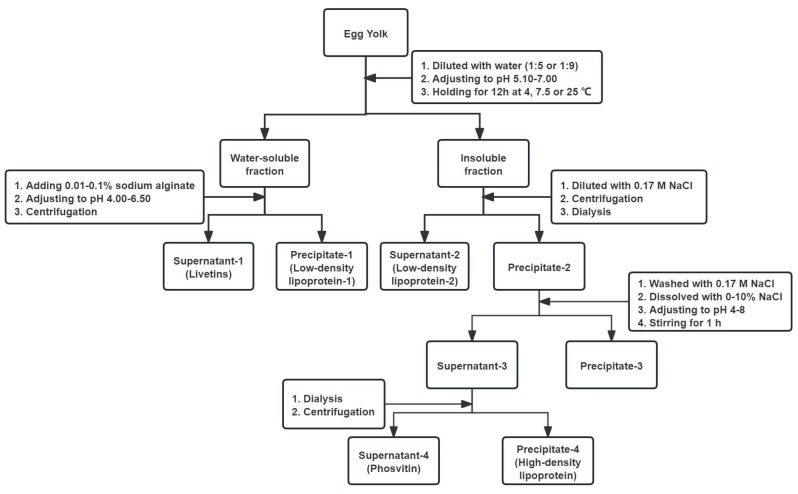
Procedure for isolation of different egg yolk components.

The procedure for the isolation of different egg yolk components is shown in Figure 1. The egg yolk was separated from broken fresh eggs and rolled on filter papers to remove egg white. The egg yolk membrane was punctured to collect the pure egg yolk.

#### 2.2.1. Separation of Water-Soluble Fraction (WSF) and Insoluble Fraction (ISF)

Different initial volumes of egg yolks were diluted to 150 mL with either 5 or 9 volumes of distilled water stirred for 1 h in an ice bath, and then held for 12 h at 4 °C, 7.5 °C, or 25 °C. Another ten aliquots of egg yolk solutions (1:9 dilution) were adjusted to pH 5.10–7.00 and held for 12 h at 4 °C. WSF and ISF were separated by decantation.

#### 2.2.2. Separation of Livetins and Low-Density Lipoprotein (LDL)

Sodium alginate solution (1%, *w*/*v*) was added to WSF to achieve a 0.01–0.1% (*w*/*v*) final concentration. Then, WSF was adjusted to pH 5.40 and stirred for 10 min. Sodium alginate was added to another ten aliquots of WSF to achieve a 0.05% (*w*/*v*) final concentration, and then WSF was adjusted to pH 4.00–6.50. After centrifuging at 4 °C for 10 min at 2500× *g*, the obtained supernatant and precipitate were named Supernatant-1 and Precipitate-1, respectively.

ISF was diluted with an equal volume of 0.17 M NaCl solution stirred for 1 h in an ice bath, and then centrifuged at 4 °C for 1 h at 10,000× *g*. The obtained Supernatant-2 was dialyzed against distilled water and lyophilized.

#### 2.2.3. Separation of Phosvitin (PV) and High-Density Lipoprotein (HDL)

The Precipitate-2 obtained from ISF was washed three times with a 0.17 M NaCl solution, followed by dissolution in 9 volumes of NaCl solutions (0–10%, *w*/*v*), with pH adjustment within the range of 4–8. The above solutions were stirred at room temperature for 1 h and centrifuged at 2500× *g* for 10 min to obtain Supernatant-3 and Precipitate-3. Then, Supernatant-3 was dialyzed against distilled water and centrifuged to obtain Supernatant-4 and Precipitate-4.

### 2.3. Sodium Dodecyl Sulfate Polyacrylamide Gel Electrophoresis (SDS-PAGE)

SDS-PAGE was carried out using the method of Zhang et al. (2022) with a 10% separating gel and a 5% stacking gel, with some modification [18]. The gels of WSF and Supernatant-1 were stained with Coomassie brilliant blue R-250. The gels of Supernatant-3 and Supernatant-4 were stained using the phosphoprotein staining method described by Hegenauer et al. (1977) [19]. The percentages of electrophoretic bands in each lane were calculated by the image lab gel system (ChemiDoc XRS+, Bio-Rad, Richmond, CA, USA) and the tests were repeated in triplicate.

### 2.4. Particle Size and Zeta-Potential

The particle size and Zeta-potential of Precipitate-2 were measured with a Malvern laser particle size analyzer (Worcestershire, UK) using a 1 cm path-length sample pool and a DTS1070 folded capillary cell.

### 2.5. The Determination of Free Ca^2+^ Concentration

An equal volume of Supernatant-3 was pipetted into an ultrafiltration centrifuge tube (3 kDa MWCO, Amicon^®^ Ultra, Merck, Darmstadt, Germany) and centrifuged at 6000× *g* for 10 min. The free Ca^2+^ concentration in the obtained filtrate was determined using a calcium content assay kit (Solarbio, Beijing, China).

### 2.6. Composition Analysis

The contents of crude protein and total lipid of egg yolk components were determined using the Kjeldahl method and the method of Folch et al. (1957), respectively [20]. The phospholipid content was obtained by measuring the phosphorus content in total lipid and then converting it using a conversion factor of 25 [21].

### 2.7. Statistical Analysis

All experiments were carried out in triplicate unless otherwise stated, and the obtained data were expressed as the means ± standard deviations. The two-way ANOVA was conducted with NaCl (0–10%) and pH (pH 4–8) as main factors and a NaCl × pH interaction term using GraphPad Prism 8.0 (San Diego, CA, USA), followed by Tukey’s post hoc test. Samples designated with different lowercase and uppercase letters were significantly different (*p* < 0.05). The analysis of the correlation between Zeta-potential, particle size, and free Ca^2+^ concentration was evaluated by Pearson’s correlation test, and * *p* < 0.05 or ** *p* < 0.01 represented different statistically significant levels.

## 3. Results

### 3.1. Effects of Dilution Ratio, Temperature, and pH on the Separation of Water-Soluble Fraction (WSF) and Insoluble Fraction (ISF)

**Figure 2:**
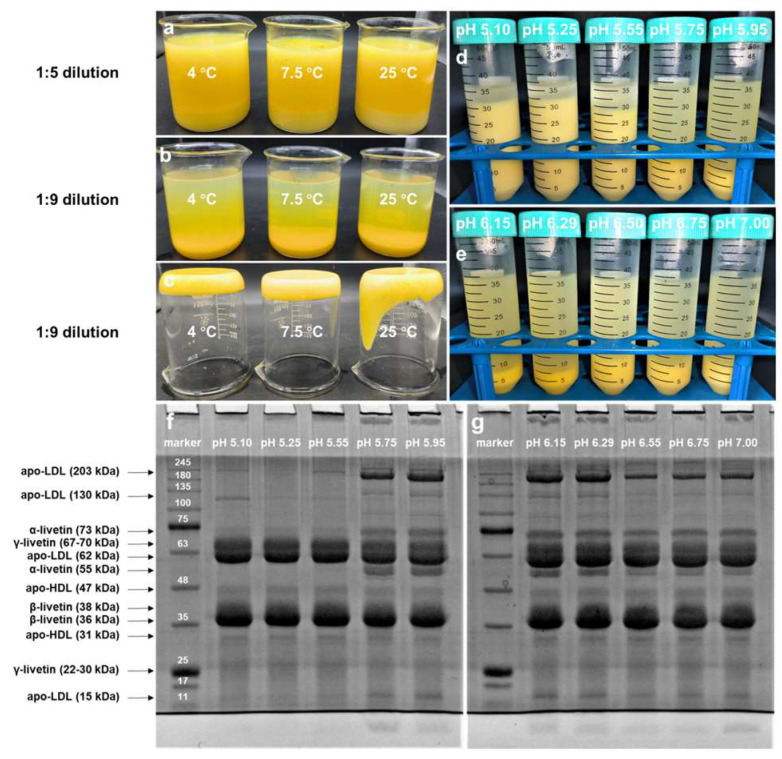
The appearance of (**a**) 1:5 diluted egg yolk; (**b**) 1:9 diluted egg yolk; (**c**) Insoluble fraction obtained from 1:9 diluted egg yolk at different temperatures; and (**d**,**e**) 1:9 diluted egg yolk (4 °C) at different pH. (**f**,**g**) SDS-PAGE of water-soluble fraction obtained from 1:9 diluted egg yolk (4 °C) at different pH.

Reducing the residue of lipoproteins in WSF is a prerequisite for livetin separation. WSF obtained at a 1:9 dilution ratio appeared clearer compared to those obtained at 1:5, indicating less lipoprotein residue (Figure 2a,b). In addition, the ISF formed at 4 °C and 7.5 °C were more compact than the one formed at 25 °C, which facilitated the separation of supernatants and thus omitted centrifugation operations (Figure 2c). This result might be attributed to the decreased dispersibility of lipids in lipoproteins at lower temperatures [22]. Those ISF were suspended in the form of loose aggregates at pH 5.10, pH 5.25, and pH 5.55 (Figure 2d). Interestingly, there was almost no residue of lipoproteins in their corresponding WSF. The heights of ISF formed at pH 6.15 and pH 6.29 (original pH) were lower (Figure 2e), while our SDS-PAGE studies indicated that WSF mainly contained low-density lipoprotein (203 kDa, 130 kDa, 62 kDa and 15 kDa), α-livetin (73 kDa and 55 kDa), β-livetin (38 kDa and 36 kDa), γ-livetin (67–70 kDa, 22–30 kDa) (Figure 2g). These results indicated that temperature and pH had a significant impact on the amount and suspension state of low-density lipoprotein in WSF. Adjusting the pH of the 1:9 diluted egg yolk to pH 6.15–6.29 and incubating it at 4–7.5 °C for 12 h could effectively separate the egg yolk WSF and ISF.

### 3.2. Effects of Sodium Alginate and pH on the Removal of Low-Density Lipoprotein (LDL) from Water-Soluble Fraction (WSF)

**Figure 3:**
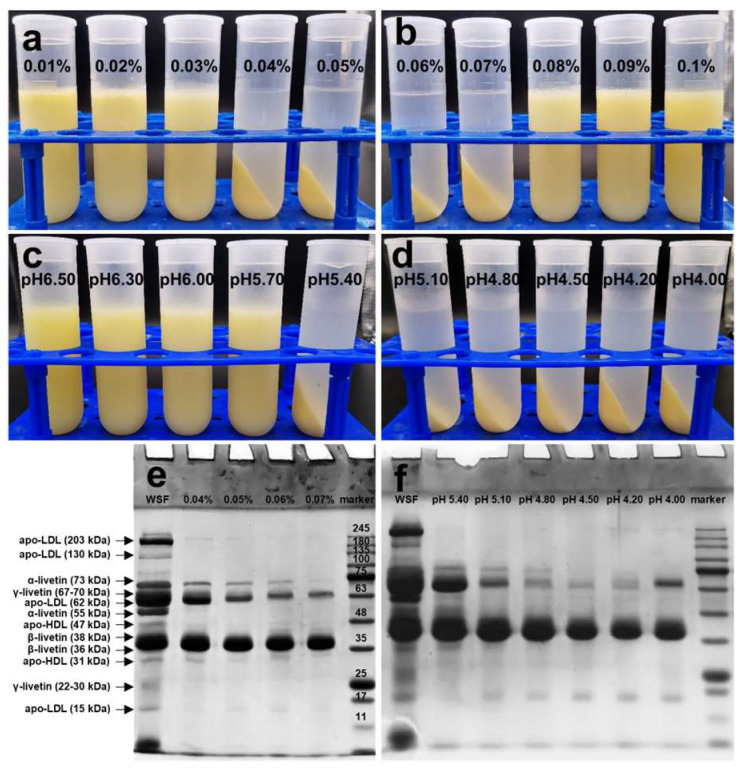
The appearance of (**a**,**b**) Water-soluble fraction with the different concentrations of sodium alginate at pH 5.40; (**c**,**d**) Water-soluble fraction with 0.05% sodium alginate at different pH. (**e**,**f**) SDS-PAGE of water-soluble fraction and Supernatant-1.

As shown in Figure 3a,b, the WSF became turbid after adding sodium alginate, and when the final concentration of sodium alginate was 0.04% to 0.07%, WSF could be separated into two phases by centrifugation. The pH was another crucial factor affecting precipitate formation and the effective pH range was pH 5.40 to pH 4.00 (Figure 3c,d). The disappearance of bands near 203 kDa, 130 kDa, 62 kDa, and 15 kDa in the SDS-PAGE of WSF indicated the removal of LDL (Figure 3e,f). The nature of interactions between proteins and polysaccharides, predominantly electrostatic but also involving hydrogen bonding, hydrophobic attractions, or steric repulsion, depends on their structure and can be significantly altered by environmental factors such as pH and ionic strength [23]. LDL could form complexes with sodium alginate through electrostatic forces at a pH lower than its isoelectric point (pH between 6 and 7) [24,25]. However, some livetins were also precipitated with decreasing pH or increasing sodium alginate concentration (Figure 3e,f). Overall, adding 0.04–0.05% sodium alginate to WSF and adjusting the pH to 5.40 might be more suitable for the effective removal of LDL. Given that livetins, phosvitin (PV), LDL, and high-density lipoprotein (HDL) collectively account for 98% of the dry matter in egg yolk, with livetins being multimeric complex proteins and LDL and HDL being lipoproteins, conventional protein yield and purity might not be suitable for evaluating the effectiveness of protein separation in this case. Instead, the crude separation effectiveness was evaluated through macroscopic images, SDS-PAGE, and analysis of lipoproteins composition in this study. As shown in Table 1, when LDL in WSF was removed in the form of a precipitate, the percentage of electrophoretic bands for livetins increased from 51.90% to 91.04%, and the percentage for γ-livetin increased from 15.80% to 33.51%.

### 3.3. Effects of pH and NaCl on the Dispersion State of Precipitate-2

**Figure 4:**
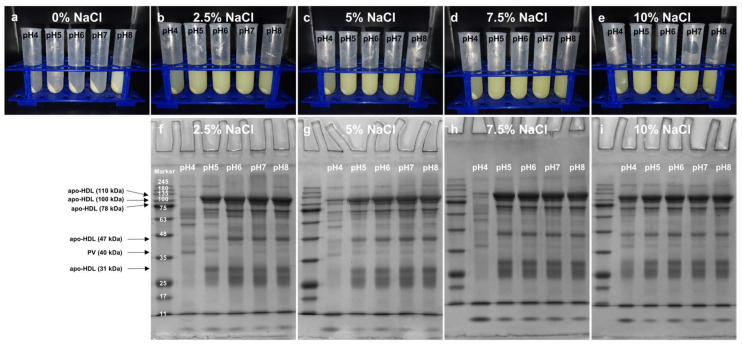
(**a**–**e**) The dispersion state of Precipitate-2 at different concentrations of NaCl and pH after centrifugation. (**f**–**i**) SDS-PAGE of Supernatant-3 obtained at different concentrations of NaCl and pH.

After washing, Precipitate-2 resembles egg yolk granules, which are supramolecular assemblies of high-density lipoprotein (HDL) and phosvitin (PV) driven by phosphocalcic bridges [26]. Changing the dispersion state of Precipitate-2 is one of prerequisites for the further separation. As shown in Figure 4a, Precipitate-2 was insoluble in water at pH 4–7 and formed two distinct phases after centrifugation, while part of Precipitate-2 was dispersed in the supernatant at pH 8. Figure 4b–e showed that Precipitate-2 could gradually disperse in the solution as the pH and NaCl concentration increased, which indicated that both NaCl and pH could impact the dispersion state of Precipitate-2. When the concentration of NaCl solution (pH 5–8) reached 5% or above, only a small amount of Precipitate-3 remained at the bottom of each tube, which could be considered as waste material (Figure 4c–e). Our SDS-PAGE studies showed that Supernatant-3 was mainly composed of HDL (110 kDa, 100 kDa, 78 kDa, 47 kDa, and 31 kDa) and PV (40 kDa) (Figure 4f–i). The changes in SDS-PAGE band intensity further confirmed that more and more HDL and PV were dispersed in Supernatant-3 with the increase in pH and NaCl concentration. However, further confirmation is needed to determine the reasons for the change in the dispersion state of Precipitate-2 and the dispersion forms of HDL and PV in Supernatant-3, either individually or as an HDL-PV complex.

### 3.4. Effects of pH and NaCl on the Zeta-Potential, Particle Size, and Phosphocalcic Bridges Breakage of Precipitate-2

**Figure 5:**
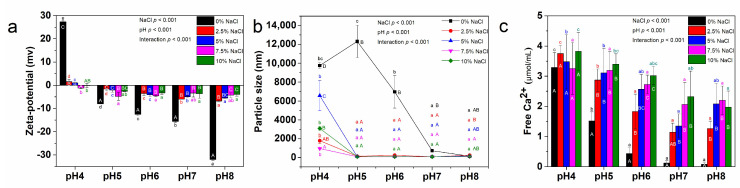
(**a**) Zeta-potential; (**b**) Particle size; (**c**) Free Ca^2+^ concentration of Precipitate-2 (Different lowercase letters in the same color indicate significant differences (*p* < 0.05) in values between different pH groups at the same NaCl concentration. Different uppercase letters indicate significant differences (*p* < 0.05) in values between different NaCl concentration groups at the same pH).

The dispersion state of Precipitate-2 is closely related to the Zeta-potential, particle size, and phosphocalcic bridge breakage. As shown in Figure 5a, the zero Zeta-potential point of Precipitate-2 was measured between pH 4 and pH 5, and the negative potential increased with increasing pH. The part dispersion of Precipitate-2 in the supernatant at pH 8 might be attributed to the increased electrostatic repulsion (Figure 4a). The addition of NaCl screened the surface charge of Precipitate-2, as evidenced by the decrease in the absolute potential of Precipitate-2. As the ionic strength and pH increased, there were decreases in particle size, indicating the disaggregation of Precipitate-2 particles (Figure 5b). The critical NaCl concentration and pH were observed to be 2.5% and pH 5, respectively, as the particle size did not change significantly beyond this point. The concentration of free Ca^2+^ decreased with increasing pH, particularly noticeable in the 0% and 2.5% NaCl groups, as further confirmed by the strong correlation between Zeta-potential and free Ca^2+^ (Figure 5c and Table 2). This result suggested that the original free Ca^2+^ in Precipitate-2 would be adsorbed on the surface of particles with increasing electronegativity. An increase in free Ca^2+^ concentration was observed with the gradual addition of NaCl at pH 5–8 groups (Figure 5c). Probably, high concentrations of sodium chloride results in the substitution of divalent calcium ions by monovalent sodium ions, thereby disrupting phosphocalcic bridges and leading to the subsequent release of calcium ions [27]. Interestingly, the free Ca^2+^ concentration in the pH 5–8 groups remained lower compared to that in the pH 4 group, even when the NaCl concentration reached 7.5% and 10%. This result might be attributed to the Ca^2+^ released from phosphocalcic bridges adsorbed onto the surface of negatively charged particles at pH 5–8, thereby participating in the shielding of surface charges.

Two-way ANOVA showed that both pH and NaCl concentration had significant effects on Zeta potential, particle size, and free Ca^2+^ concentration, and there was a significant interaction between the two factors (Figure 5). Li et al. (2022) also reported that NaCl has pH-dependent Janus effects on the structure of egg yolk granules [28]. It seemed that the impact of NaCl addition at different pH on the dispersion state of Precipitate-2 extended beyond the disruption of phosphocalcic bridges. When the artificially added Na^+^ and the released Ca^2+^ (hydration number 4 for Na^+^ and 6 for Ca^2+^) adsorbed on the surface of the particles, Precipitate-2 achieved colloidal stability under strong hydration repulsion [29]. The strong correlation among the three indicators was well demonstrated by the pH 6 (0–10% NaCl) (Table 2). The alteration in the potential of amphiphilic proteins induced by pH regulation is crucial in determining whether highly hydrated Na^+^ and Ca^2+^ can be adsorbed onto the particles. Sufficient hydration repulsion is challenging to achieve when Cl^−^ (hydration number 1) adheres to the positively charged surface of proteins. Hence, even in a 10% NaCl solution, Precipitate-2 dispersion at pH 4 remained inadequate (Figure 4e). It is evident that good dispersibility of Precipitate-2 facilitates Na^+^-induced disruption of phosphocalcic bridges.

### 3.5. Effects of Dialysis on the Removal of High-Density Lipoprotein (HDL) from Supernatant-3

**Figure 6:**
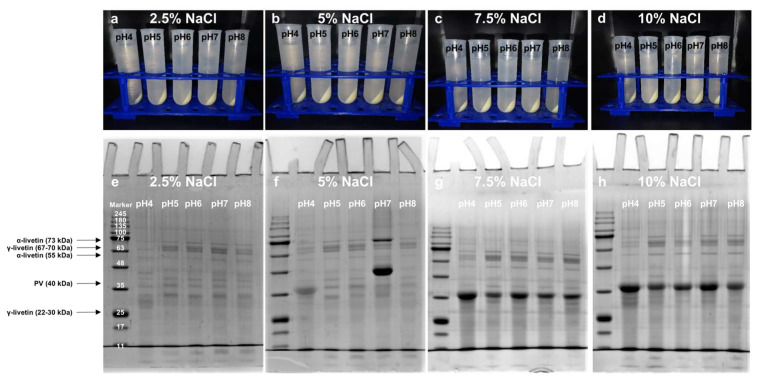
(**a**–**d**) The appearance of the dispersion state of Supernatant-3 after dialysis and centrifugation. (**e**–**h**) SDS-PAGE of Supernatant-4.

After NaCl was removed by dialysis, the Supernatant-3 could be separated into two phases again by centrifugation, even in the 7.5% and 10% NaCl groups where phosphocalcic bridges were severely destroyed (Figure 6). It suggested that the insolubility of the HDL-PV complex primarily stemmed from the inherent insolubility of HDL itself at neutral pH, rather than the presence of phosphocalcic bridges. The SDS-PAGE of Supernatant-4 obtained from 5% NaCl groups also confirmed this speculation (Figure 6f). Although Precipitate-2 could disperse well in 5% NaCl solution at pH 5–8, the lower PV band intensity suggested that the disruption of phosphocalcic bridges was not sufficient (Figure 4c and Figure 6f). As shown in Figure 6g,h, phosphocalcic bridges were fully disrupted when the concentration of NaCl reached 7.5% and above, and PV was released and dissolved in Supernatant-4. At both 7.5% (pH 7) and 10% NaCl (pH 7), the percentage of electrophoretic bands of PV in Supernatant-3 was approximately 8.5% (Table 1). Upon the removal of HDL as a precipitate, the percentage of electrophoretic bands of PV in supernatant-4 increased to 44.84% and 51.02% (Table 1). An analysis of electrophoretic bands showed that the primary impurities in the crude PV were soluble livetins (Figure 6 and Table 1). It can be further removed by increasing the washing cycles for Precipitate-2 and the volume of 0.17 M NaCl. Although the purity of PV obtained at pH 4 was higher compared with that obtained at pH 7, due to the poor dispersibility of Precipitate-2 at pH 4, more PV and HDL were retained in Precipitate-3 rather than dissolved in Supernatant-3 (Figure 4d,e). Overall, dialysis following treatment with 7.5% or 10% NaCl is an effective method for HDL removal, while the selection of pH can be adjusted according to the desired purity and yield of PV.

### 3.6. Characterization of Low-Density Lipoprotein (LDL) and High-Density Lipoprotein (HDL) as By-Products in the Crude Separation of Livetins and Phosvitin (PV)

Egg yolk LDL has a typical lipoprotein micellar structure, characterized by a neutral lipid core surrounded by an outer layer of phospholipids and apoproteins [30]. It finds widespread use in food emulsion preparation and nutrient delivery [31]. LDL is composed of about 12% of proteins and 87% of lipids, and it is this unique composition that imparts its excellent emulsifying properties [30]. Therefore, the basic properties of Precipitate-1 (LDL-1) and Supernatant-2 (LDL-2) were further determined. As shown in Table 3, the composition of LDL-1 and LDL-2 were similar to those of LDL obtained through 40% saturation of ammonium sulfate precipitation [30]. The main fatty acids in LDL-1 and LDL-2 were oleic acid (C18:1), palmitic acid (C16:0), linoleic acid (C18:2), and stearic acid (C18:0) (Table 4). Egg yolk HDL was originally obtained and defined by Joubert and Cook (1958) by extraction with MgSO_4_ and NaCl and then ultracentrifuged at 105,000× *g* for 2 h [32]. HDL is made up of 75–80% proteins and 20–25% lipids (of which phospholipids constitute 65%), making it an excellent nutritional component [33]. The total lipid content of Precipitate-4 (HDL) was higher, whereas the proportion of phospholipids was lower (Table 3). It might be attributed to the contamination of LDL and the loss of amphiphilic phospholipids during washing and subsequent separation, respectively. Residual LDL could be avoided by increasing the centrifugal force and time during Supernatant-2 and Precipitate-2 acquisition. Just as previously reported, the fatty acid compositions of egg yolk HDL and LDL were similar (Table 4) [34]. It appears that the above separation operation has no significant effect on the fatty acid composition of HDL either.

## 4. Discussion

According to OECD Agriculture statistics (database), the least squares growth rate of egg production from 2013 to 2022 is 3.01%, while it is estimated to be only 1.12% from 2023 to 2032 [35]. The separation of high-value-added livetins and phosvitin (PV) from egg yolk is a key strategy to mitigate the imbalance in demand between egg whites and egg yolk, thereby promoting the sustainable development of the egg industry [3]. Although methods for obtaining high-purity livetins and PV have been established, reducing separation costs through appropriate pretreatment remains a key challenge in practical production. A traditional route for egg yolk fractionation begins with mixing the yolk with an equal volume of 0.17 M NaCl, followed by centrifugation at 10,000× *g*, which separates the mixture into plasma (approximately 85% low-density lipoprotein (LDL) and 15% livetins) and granules (approximately 70% high-density lipoprotein (HDL), 16% PV, and 12% LDL) [36]. However, a drawback of this route is the introduction of NaCl at the initial stage of separating the plasma and granules. It means that dialysis operations need to be performed in all egg yolk protein separation processes. Therefore, this study employed a water dilution method based on solubility differences. Our separation route avoids the introduction of NaCl during the livetin separation process.

As described in Section 3.1, dilution ratio, temperature, and pH are key factors affecting the separation of water-soluble fraction (WSF) and insoluble fraction (ISF). Adjusting the pH of the 1:9 diluted egg yolk to pH 6.15–6.29 and incubating it at 4–7.5 °C for 12h effectively minimized the inclusion of lipoproteins in the WSF, facilitated the formation of a more compact ISF, and eliminated the requirement for centrifugation. Previous researchers precipitated lipoproteins by directly adding natural gums or anionic polysaccharides to diluted egg yolk or plasma, which could potentially hinder further separation of residue [24,37,38,39]. In this study, sodium alginate was added to WSF mainly composed of livetins and partial LDL. Due to a portion of LDL (Supernatant-2) remaining in the ISF, the amount of sodium alginate added to the WSF will be less than that added directly to egg yolk or plasma. Simultaneously, this selective addition also prevents contamination of other egg yolk components and alterations of their physicochemical properties. Adding 0.04–0.05% sodium alginate to WSF and adjusting the pH to 5.40 effectively improved the electrophoretic purity of livetins and γ-livetin (Table 1). The centrifugal removal of LDL-1 (Precipitate-1) also provides a pathway for continuous production in the subsequent purification of livetins through membrane separation. The obtained LDL-1 (Precipitate-1) and LDL-2 (Supernatant-2) are similar in composition to LDL isolated through ammonium sulfate precipitation (Table 3), and have potential applications in cryoprotection and nutrient delivery [40,41].

The insolubility of the HDL-PV complex was previously attributed to the presence of phosphocalcic bridges between them; however, in this study, we speculate that it may be due to the inherent insolubility of HDL at neutral pH. This speculation may alter the subsequent separation procedure of HDL and PV. In previous studies, 15% (*w*/*v*) of (NH_4_)_2_SO_4_ [42] and 3% polyethylene glycol (PEG6000) [43] were used to further separate HDL and PV co-dissolved in high-concentration NaCl solution. PV possesses a considerable quantity of negative charges and an unusually low proportion of nonpolar hydrophobic side chains, making it highly hydrophilic [6]. Therefore, PV and HDL can be further separated based on their solubility differences in low ionic strength solutions at neutral pH without the involvement of neutral salts and polymers. This adjustment will omit the use of expensive processing aids, such as (NH_4_)_2_SO_4_ and PEG6000. Although some phospholipids were lost during the washing process, the proportion of phospholipids in the total lipids of Precipitate-4 (HDL) remained at 42.05%. In short, this study attempts to reduce operational units and the use of more types of processing aids based on the differences in solubility, dispersion state, and density among egg yolk proteins under various conditions.

## 5. Conclusions

In this study, the crude separation of egg yolk proteins was based on the differential properties of proteins under various factors. The dispersibility of lipoproteins decreased at the original pH under lower temperature conditions, facilitating the formation of clear water-soluble fraction (WSF) and compact insoluble fraction (ISF) following incubation of diluted egg yolk. Low-density lipoprotein (LDL) in WSF could form a precipitate with sodium alginate through electrostatic interaction after adjusting pH and then be separated from livetins. The dispersion state and the breakage of phosphocalcic bridges of the high-density lipoprotein–phosvitin (HDL-PV) complex could be controlled by adjusting the NaCl concentration and pH. The strong hydration repulsion provided by Na^+^ and Ca^2+^ adsorbed on the protein surface was the key factor in changing the dispersibility of HDL. The inherent insolubility of HDL at low ionic strength and neutral pH allowed HDL and PV to be separated by dialysis followed by centrifugation. In summary, the above process achieved the co-production of livetins and PV by jointly adjusting temperature, pH, and ionic strength while only introducing two processing aids, sodium alginate and NaCl.

## Figures and Tables

**Table 1:** The percentage of electrophoresis bands for livetins, γ-livetin, and phosvitin in different fractions.

Fractions	Livetins (%)	γ-Livetin (%)	Phosvitin (%)
Water-soluble fraction (1:9 dilution, 4 °C, pH 6.29)	51.90 ± 0.63 ^a^	15.80 ± 0.71 ^a^	-
Supernatant-1 (0.05% sodium alginate, pH 5.40)	91.04 ± 2.83 ^b^	33.51 ± 1.55 ^b^	-
Supernatant-3 (7.5% NaCl, pH 7)	13.08 ± 0.90 ^A^	-	8.45 ± 0.24 ^A^
Supernatant-3 (10% NaCl, pH 7)	12.87 ± 0.52 ^A^	-	8.49 ± 0.71 ^A^
Supernatant-4 (7.5% NaCl, pH 4)	29.92 ± 1.25 ^B^	-	60.99 ± 0.71 ^D^
Supernatant-4 (7.5% NaCl, pH 7)	42.46 ± 0.84 ^D^	-	44.84 ± 1.83 ^B^
Supernatant-4 (10% NaCl, pH 4)	30.45 ± 1.96 ^B^	-	61.50 ± 2.00 ^D^
Supernatant-4 (10% NaCl, pH 7)	37.83 ± 1.03 ^C^	-	51.02 ± 1.37 ^C^

Note: Different lowercase letters indicate significant differences (*p* < 0.05) in values between Water-soluble fraction and Supernatant-1. Different uppercase letters indicate significant differences (*p* < 0.05) in values between Supernatant-3 and Supernatant-4.

**Table 2:** Pearson correlation coefficient of Zeta-potential, particle size, and free Ca^2+^ concentration.

	Zeta-Potential × Particle Size	Zeta-Potential × Free Ca^2+^	Particle Size × Free Ca^2+^
0% NaCl (pH 4–8)	-	0.9460 *	-
2.5% NaCl (pH 4–8)	-	0.9821 **	-
5% NaCl (pH 4–8)	-	-	-
7.5% NaCl (pH 4–8)	0.9329 *	-	-
10% NaCl (pH 4–8)	0.9417 *	-	-
pH 4 (0–10% NaCl)	-	-	-
pH 5 (0–10% NaCl)	−0.8842 *	-	−0.9687 **
pH 6 (0–10% NaCl)	−0.9906 **	0.8930 *	−0.9146 *
pH 7 (0–10% NaCl)	−0.9748 **	0.9174 *	-
pH 8 (0–10% NaCl)	-	0.9396 *	-

* *p* < 0.05; ** *p* < 0.01.

**Table 3:** The composition of egg yolk components.

	Crude Protein Content (%)	Total Lipid Content (%)	Proportion of Phospholipids in Total Lipid (%)
Precipitate-1 (LDL-1)	20.03 ± 0.36	74.11 ± 2.34	24.63 ± 1.26
Supernatant-3 (LDL-2)	11.67 ± 0.21	85.79 ± 1.06	27.29 ± 1.67
Precipitate-4 (HDL)	67.45 ± 0.43	30.35 ± 4.36	42.05 ± 6.42

**Table 4:** Fatty acid composition of egg yolk components.

Fatty Acid	Precipitate-1 (LDL-1)	Supernatant-2 (LDL-2)	Precipitate-4 (HDL)
C14:0	0.31 ± 0.00	0.38 ± 0.00	0.30 ± 0.00
C14:1	0.07 ± 0.00	0.09 ± 0.00	-
C15:0	0.05 ± 0.00	0.05 ± 0.00	0.06 ± 0.00
C15:1	-	-	0.06 ± 0.01
C16:0	25.96 ± 0.01	25.82 ± 0.03	26.74 ± 0.03
C16:1	3.45 ± 0.01	3.31 ± 0.00	3.28 ± 0.01
C17:0	0.12 ± 0.00	0.13 ± 0.01	0.13 ± 0.01
C17:1	0.11 ± 0.01	0.13 ± 0.01	0.10 ± 0.01
C18:0	7.90 ± 0.00	8.21 ± 0.01	9.63 ± 0.02
C18:1	44.67 ± 0.05	44.68 ± 0.02	41.00 ± 0.02
C18:2	14.16 ± 0.02	13.78 ± 0.02	13.84 ± 0.01
C18:3n6	0.09 ± 0.00	0.10 ± 0.00	0.09 ± 0.00
C18:3n3	0.35 ± 0.00	0.38 ± 0.00	0.27 ± 0.00
C20:0	0.02 ± 0.00	0.01 ± 0.01	0.02 ± 0.00
C20:1	0.18 ± 0.01	0.16 ± 0.01	0.19 ± 0.01
C20:2	0.17 ± 0.01	0.14 ± 0.03	0.17 ± 0.01
C20:4	1.78 ± 0.00	1.86 ± 0.00	3.12 ± 0.01
C22:6	0.59 ± 0.04	0.79 ± 0.02	-
C24:0	-	-	1.00 ± 0.01

## Data Availability

The original contributions presented in the study are included in the article, further inquiries can be directed to the corresponding author.
